# Does Loneliness Necessarily Lead to a Decrease in Prosocial Behavior? The Roles of Gender and Situation

**DOI:** 10.3389/fpsyg.2016.01388

**Published:** 2016-09-16

**Authors:** Heqing Huang, Yanchun Liu, Xiaocen Liu

**Affiliations:** ^1^School of Early Childhood Education, Capital Normal UniversityBeijing, China; ^2^Youth Work Department, China Youth University of Political StudiesBeijing, China

**Keywords:** loneliness, loneliness-perpetuation perspective, loneliness-reduction perspective, prosocial behavior, gender difference

## Abstract

Although, previous studies show overwhelming evidence that loneliness is negatively correlated with prosocial behavior, some theories and research have implied that under certain situations, loneliness plays a positive role in an individual's social functioning. The two studies reported in this article examined loneliness and its associations with prosocial behavior in Chinese adults using subjective reporting and experimental design. Study 1 examined 305 Chinese adults (175 males) using the *Social and Emotional Loneliness Scale for Adults* and the *Prosocial Tendencies Measure* to evaluate their loneliness and prosocial tendencies. The results showed that loneliness was negatively associated with all prosocial tendencies except the public prosocial tendency. Study 2 examined 177 Chinese adults (61 males) using an experimental design and found that only lonely women in public situations expressed a greater willingness to help. The results also suggest that loneliness may play a positive role in the social functioning of individuals under certain conditions. The function of loneliness and the implications of the association between loneliness and prosocial behavior are discussed.

## Introduction

Prosocial behavior represents a broad category of acts that are defined as generally beneficial to other people (Penner et al., 2005). The attempts to understand the economic and psychological motivations for prosocial behavior date back several hundred years, and there are still continuing debates concerning the nature of prosocial behavior. There are generally two categories of theories and models (Batson et al., 1989). Generally speaking, there are competing motives for prosocial behavior, especially charitable giving, including pure altruism and egoistic motivation. People may help others out of a pure altruistic motivation when there is no internal or external reward for giving or helping people. As the empathy-altruism hypothesis claims, empathy (the feelings of compassion, sympathy, tenderness) evokes an altruistic motive, the ultimate goal of which is to protect or promote the welfare of the person for whom empathy is felt (Batson et al., 1989). In contrast, egoistic motivation may come from a desire to win prestige, respect, friendship, and other social and psychological objectives (Omoto and Snyder, 1995). From this perspective, humans are relatively rational and primarily concerned about their self-interest. Moreover, Andreoni (1990) proposes an economic phenomenon, which he calls “warm-glow giving.” There is a trade-off between the two motivations in any situation, the characters of which may affect the degree of the trade-off.

Loneliness is the subjective experience of dissatisfaction with one's social-relational life (e.g., Shevlin et al., 2014). Loneliness is such a painful experience that people will do practically anything to avoid it, and consequently loneliness may have repercussions for social functioning, including prosocial behavior. According to Vanhalst et al. (2015), there are two conflicting perspectives on how people respond when the fundamental needs to belong are not met. The first is labeled as the *loneliness-perpetuation perspective*, which posits that loneliness reduces sensitivity to the potential benefits of situations that may satisfy the need to belong. A lot of previous research has demonstrated the detrimental effect of loneliness on an individual's social functioning (Salovey et al., 1991; Cassidy and Asher, 1992; Twenge et al., 2007; Woodhouse et al., 2012).

The second is labeled as the *loneliness-reduction perspective*, which posits that a frustrated need to belong provides an impetus for individuals to actively seek to reduce need frustration and to increase need satisfaction. There are some studies that give support to the *loneliness-reduction perspective*. For example, lonely participants are more likely to remember the information related to interpersonal or collective social ties (Gardner et al., 2005) or show greater attention to emotional vocal tone (Nathan et al., 2009) than their non-lonely counterparts. The lonely individuals even tend to exhibit physical warmth-seeking behavior (Shalev and Bargh, 2015). Moreover, a greater sense of loneliness is associated with a stronger communal orientation and less shyness (Clark et al., 2015). In sum, the lonely individuals seem to have more motivation to reconnect with others.

However, it is not clear whether there are competing effects of loneliness on prosocial behavior, which is one of the most important social functions. Almost all empirical research has found that loneliness has a detrimental effect on an individual's prosocial behavior. The negative correlations between loneliness and prosocial behavior have been found in various developmental stages, such as childhood (Cassidy and Asher, 1992; Zysberg, 2012), adolescence (Woodhouse et al., 2012), and adulthood (Carlson et al., 1988; Williamson and Clark, 1989; Salovey et al., 1991). Because prosocial behavior is also a means of connecting with others (Penner et al., 2005), it is reasonable to assume that loneliness is positively correlated with prosocial behavior in certain situations. However, such effects of loneliness have not been demonstrated thus far. It may be because previous studies did not investigate prosocial behavior taking place in different situations. Whether prosocial behavior takes place in public or in private may play an important role in the relationship between loneliness and prosocial behavior. When witnesses are lacking, prosocial behavior cannot help the lonely individual reconnect with other people; the individual's need to belong may reduce to avoid the painful feeling of loneliness; and consequently his/her tendency toward prosocial behavior is likely to diminish. Therefore, in the private situation, the *loneliness-perpetuation perspective* may be more appropriate. In the public situation, however, where prosocial behavior can be witnessed by others, the individual will be motivated by the need to belong to do something to gain appreciation and a reputation. Therefore, in the public situation, the *loneliness-reduction perspective* may be more appropriate.

Moreover, gender may also affect the relationship between loneliness and prosocial behavior. More and more research has demonstrated that there are different ways of mental processing in males and females (Maccoby and Jacklin, 1974; Francis and Fallon, 2005), and the gender difference was found not only in loneliness (Deniz et al., 2005; Knox et al., 2007) but also in its relationship with social functioning (Hanson and Jones, 1981; Haferkamp et al., 2012; Gohier et al., 2013). Thus, the present study also aims to examine the role of gender in the relationship between loneliness and prosocial behavior.

In sum, the aim of the present study is to examine the effect of loneliness on prosocial behavior and the potential mediating factors such as situation and gender. Based on the previous theoretical and empirical research, we propose that the relationship between loneliness and prosocial behavior may vary across different situations, and be moderated by gender.

## Study 1

As Carlo and Randall (2002) indicated, prosocial behavior is a complex construct and consists of six dimensions that are driven by six different motivations. We hypothesized that trait loneliness would be differentially associated with various prosocial tendencies.

### Materials and methods

#### Participants

The participants included 305 undergraduate students (130 females and 175 males) who attended part-time courses at a university in northern China. The ages of the participants ranged from 23 to 37 years (*M* = 27.45, *SD* = 3.04). Active informed consent was obtained from all participants, and they were treated in accordance with the American Psychological Association's ethical principles.

#### Materials and procedure

The participants were given the two questionnaires, as described below, in a classroom setting. They completed the measures in group sessions that took no longer than 30 min. To avoid social desirability effects, the titles of the scales were not displayed, and the participants were only required to write down their student IDs.

##### Loneliness

A Chinese variant of the Social and Emotional Loneliness Scale for Adults (SELSA-C) was used to assess loneliness among adults, derived from those of DiTommaso and Spinner (1993). The SELSA-C (Yang and Wang, 2010) is a self-reporting questionnaire that is composed of two 5-item subscales: the social loneliness subscale (e.g., “I don't have any friends who share my views, but I wish I did”) and the emotional loneliness subscale (e.g., “I wish I had a more satisfying romantic relationship”). Using a five-point Likert scale ranging from 1 (strongly disagree) to 5 (strongly agree), the participants were asked to report the extent to which these statements apply to them. The internal reliability of the ESLSA-C and its subscales is shown in Table 1.

**Table 1:** **Descriptive statistics for the major variables and comparison of the means in Study 1**.

	**Cronbach's α**	**Total**	**Males**	**Females**	**Gender difference *t*-test**	**Cohen's *d***
Loneliness	0.77	2.80 (0.95)	2.93 (0.97)	2.63 (0.90)	*t*_(298)_ = −2.86^**^	0.321
Prosocial behavior	0.93	3.60 (0.60)	3.57 (0.60)	3.62 (0.59)	*t*_(290)_ = −0.69	−0.08
Public	0.84	2.89 (0.87)	2.83 (0.90)	2.94 (0.84)	*t*_(290)_ = −1.06	−0.13
Anonymous	0.85	3.60 (0.79)	3.51 (0.80)	3.68 (0.78)	*t*_(291)_ = −1.73	−0.22
Altruism	0.75	3.95 (0.72)	3.94 (0.66)	3.95 (0.76)	*t*_(290)_ = −0.04	−0.01
Compliant	0.83	3.76 (0.70)	3.73 (0.69)	3.78 (0.70)	*t*_(291)_ = −0.55	−0.07
Emotional	0.82	3.55 (0.72)	3.57 (0.74)	3.54 (0.72)	*t*_(291)_ = 0.39	0.04
Dire	0.76	3.83 (0.78)	3.82 (0.78)	3.84 (0.77)	*t*_(291)_ = −0.30	−0.03

** p < 0.01.

##### Prosocial tendencies

A Chinese variant (PTM-C; Kou et al., 2007) of the Prosocial Tendencies Measure (PTM; Carlo and Randall, 2002) was used to assess prosocial tendencies among Chinese adults. The PTM was originally developed to assess the self-reporting of six types of prosocial behavior among college individuals (Carlo and Randall, 2002). The six types of prosocial behavior in the PTM-C are labeled as public, anonymous, dire, emotional, compliant, and altruistic. Public prosocial behaviors are defined as behaviors that are intended to benefit others and are enacted in the presence of others (four items; e.g., “I can help others best when people are watching me”). Anonymous prosocial behaviors are defined as the tendency to help others without other people's knowledge (five items; e.g., “I think that helping others without them knowing is the best type of situation”). Dire prosocial behaviors refer to helping others under emergency or crisis situations (three items; e.g., “I tend to help people who are in real crisis or need”). Emotional prosocial behaviors are intended to benefit others and are enacted under emotionally evocative situations (five items; e.g., “I respond to helping others best when the situation is highly emotional”). Compliant prosocial behaviors refer to helping others when asked to do so (two items; e.g., “When people ask me to help them, I don't hesitate”). Altruism refers to helping others when there is little or no perceived potential for a direct, explicit reward for the helper (six items; e.g., “I often help even if I don't think I will get anything”). The items are rated on a 5-point Likert scale ranging from 1 (strongly disagree) to 5 (strongly agree). The internal reliabilities of the PTM-C and its subscales are also shown in Table 1.

### Results

The results of the descriptive analyses for males, females, and the total sample are presented in Table 1. In addition to the descriptive analyses, two other sets of analyses were conducted. First, we examined the gender difference in the ESLSA-C, the PTM-C, and their subscales (see Table 1) using a series of independent *t*-tests. Three correlation analyses were then conducted with females, males, and the entire sample to determine whether and how gender affected the correlative pattern between loneliness and prosocial tendencies (see Table 2).

**Table 2:** **Correlation between the subscales of loneliness and prosocial trend measures in Study 1**.

		**Public**	**Anonymous**	**Altruism**	**Compliant**	**Emotional**	**Dire**
Total	Loneliness	0.08	−0.18^**^	−0.29^**^	−0.13^*^	−0.18^**^	−0.25^**^
	Public		0.20^**^	0.14^*^	0.36^**^	0.43^**^	0.28^**^
	Anonymous			0.74^**^	0.59^**^	0.59^**^	0.69^**^
	Altruism				0.64^**^	0.59^**^	0.70^**^
	Compliant					0.64^**^	0.67^**^
	Emotional						0.65^**^
	Dire						1
Females	Loneliness	0.23^**^	−0.24^**^	−0.38^**^	−0.16^*^	−0.17^*^	−0.31^**^
	Public		0.20^**^	0.12	0.33^**^	0.40^**^	0.21^**^
	Anonymous			0.75^**^	0.62^**^	0.61^**^	0.68^**^
	Altruism				0.59^**^	0.60^**^	0.73^**^
	Compliant					0.61^**^	0.64^**^
	Emotional						0.61^**^
	Dire						1
Males	Loneliness	−0.14	−0.14	−0.17	−0.12	−0.18^*^	−0.18^*^
	Public		0.19^*^	0.18^*^	0.40^**^	0.47^**^	0.37^**^
	Anonymous			0.75^**^	0.56^**^	0.58^**^	0.70^**^
	Altruism				0.70^**^	0.58^**^	0.67^**^
	Compliant					0.69^**^	0.72^**^
	Emotional						0.71^**^
	Dire						1
Fisher *r*-to-*z* transformation	*Z*	3.21^*^	−0.89	−0.35	−0.09	−1.18^*^	−1.95^*^

* p < 0.05.

** p < 0.01.

First, a series of independent *t*-tests was conducted to examine the gender difference in the ESLSA-C, the PTM-C, and their subscales. As Table 1 shows, females generally feel lonelier than males. Moreover, no gender difference was found for the PTM and its six subscales.

Second, *Pearson* correlation analyses were conducted to determine the gender-specific pattern in the relationship between loneliness and prosocial tendencies. As Table 2 shows, in the full sample, loneliness negatively correlated with five subscales of prosocial tendencies but has no relationship with the public prosocial tendency. Moreover, the correlative patterns differed between males and females. In females, a public prosocial tendency was positively correlated with loneliness, and the other five subscales of prosocial tendencies were negatively correlated with loneliness. However, the results revealed a distinct pattern in males, in which loneliness had a zero or negative correlation with all six subscales of prosocial tendencies. Furthermore, we used the Fisher *r*-to-*z* transformation to calculate a value of *z* that can be applied to assess the significance of the difference between two correlation coefficients found in the male and female samples, and the *z-*values are listed in Table 2. The results indicated that loneliness and public prosocial behavior are significantly more correlated in females than in males, whilst loneliness and both the dire and the altruistic prosocial tendency are significantly less correlated in females than in males.

### Discussion

Study 1 found that the lonelier people self-reported less prosocial behavior, with the exception of one type of prosocial behavior: helping others in public. Moreover, this tendency was especially true for females.

To begin with, the negative relations with most kinds of prosocial tendencies were in accordance with previous studies in finding that loneliness, as a negative emotion that causes painful feelings, is negatively correlated with an individual's tendency to engage in prosocial behavior (Carlson et al., 1988; Williamson and Clark, 1989; Salovey et al., 1991; Cassidy and Asher, 1992; Twenge et al., 2007; Woodhouse et al., 2012). This result supports the *loneliness-perpetuation perspective* (Vanhalst et al., 2015).

Moreover, we also found that loneliness was not necessarily negatively correlated with every type of prosocial tendency. In the full sample, the public prosocial behavior bears no relationship to the full scale of loneliness. However, further examination revealed a positive relationship between loneliness and the public prosocial tendency in women. This effect supports the *loneliness-reduction perspective*. These results suggest that loneliness may be a signal of one's social disadvantage (Cacioppo et al., 2014), and a person who feels lonely will be motivated to repair and improve his/her social acceptance.

Finally, Study 1 also revealed a gender-specific correlative pattern between loneliness and the public prosocial tendency. Only in females was loneliness positively correlated with the public prosocial behavior tendency; whereas loneliness was not correlated with the males' public prosocial behavior. These results imply that loneliness may affect the prosocial behavior of males and females in different ways.

Despite the clear pattern of effects emerging from Study 1, the study design was cross-sectional and correlative, and therefore the results cannot confirm the direction of the hypothesized effect and cannot eliminate the possibility that unmeasured variables associated with loneliness could explain the relationships observed. Accordingly, an experiment was needed to examine whether in real life, the situations and feelings of loneliness affect what an individual will do when exposed to others' needs. We expect that whether or not the participants feel loneliness and whether the situation is witnessed by others will affect the individual's willingness to conduct prosocial behavior, and we also expect that the effect will be more obvious in females than in males.

## Study 2

In Study 2, we aimed to clarify the causal processes indicated in Study 1 by using an experimental design in which loneliness and public prosocial behaviors were manipulated to assess the effects on the willingness to offer time and financial help to a person in need. Consistent with the previous theory and research findings, we predicted that: (a.) the participants in the public condition would report a greater willingness to help than would the participants in a private condition; (b.) the participants in the lonely condition would report a greater willingness to help than would the participants in the non-lonely condition; and (c.) the effect of the experimental condition on the willingness to offer help would be moderated by the participants' genders.

### Methods

#### Participants and design

A total of 177 undergraduate students (61 males and 116 females) at a university in northern China participated voluntarily in the experiment in exchange for course credit. The experiment consisted of a 2 (loneliness: lonely vs. non-lonely) × 2 (prosocial situation: public vs. private) mixed design, and both loneliness and prosocial situations were between-participants factors. The participants were randomly assigned to the lonely and private (*n* = 36), lonely and public (*n* = 47), non-lonely and private (*n* = 41), and non-lonely and public (*n* = 53) conditions. The random assignment of participants to each condition was important to minimize the possibility that our effects could be explained by variables associated with loneliness and public prosocial behaviors. However, because of the random assignment and because some of the participants did not complete all the questions, the *n* values were different across different statistical analyses.

#### Procedure

After the participants' regular class, an experimenter entered the classroom and claimed that she was a staff member from the foundation in the university and that she had come to raise money and ask for volunteers to help a student who suffered from a serious disease. She asked the participants to read a test booklet carefully and answer the questions in it. During this process, the participants were divided into two groups and each group was primed for the feelings of loneliness and non-loneliness by viewing a set of priming pictures in the booklet. Then they read a collection notice which called for donations to a sick student. At this step, half of the participants were asked to donate in the public situation while the other half were asked to donate in the private situation. Because the foundation in the university often carries out surveys of the university students when collecting donations, it was not unusual that the donation collection was accompanied with the questionnaires. The participants were debriefed after the procedure, and were rewarded with a little gift for their participation. This study was approved by the Ethics and Human Protection Committee in the university and was conducted in accordance with the ethical standards laid down in the 1964 Declaration of Helsinki.

#### Materials

##### Priming for loneliness or non-loneliness

The participants first received a test booklet that was ostensibly the same, although the participants were actually randomly allocated into four conditions. In the test booklet, the participants first needed to finish a task adapted from the task of Hicks et al. (2010) to prime the feelings of loneliness and non-loneliness. The participants had to view six pictures and were asked to write down the meaning expressed by each picture. The participants in the lonely and non-lonely conditions viewed different pictures. The participants in the lonely condition viewed pictures describing a person or character in a lonely, friendless situation; the participants in the non-loneliness condition viewed pictures describing a couple, family, or team. In the present study, all participants in the lonely condition wrote words such as loneliness, isolation, alone, abandoned, and outcast, whereas all participants in the non-lonely condition wrote words such as warm, united, supported, and companionship.

##### Donation story

In the second step, all participants encountered a “true” story concerning a university student who suffered from a serious disease and urgently needed help. The participants needed to determine how much money or time he or she wanted to donate to the victim.

##### Priming for privacy or publicness

In the third step, the participants were randomly assigned to the private or public condition. In the private condition, the participants read the following text: “We hope you can help the student by donating your time and money. The decision and the amount of your donation will be strictly confidential.” The participants in the public condition read the following text: “We hope you can help the student by donating your time and money. The decision and the amount of your donation will be released on the university website, and you will get a certificate for your kindness.”

##### Willingness to help

The participants were then asked to respond to two questions (“To what extent would you be willing to offer financial help to the student?” and “To what extent would you be willing to offer your time to help the student?”) using two 5-point scales (1 = 0 RMB; 2 = 50 RMB; 3 = 100 RMB; 4 = 200 RMB; 5 = 300 RMB; 1 = 0 day; 2 = 0.5 day; 3 = 1 day; 4 = 1.5 days; 5 = 2 days, respectively). As the amount of money and time was significantly correlated (*r* = 0.388, *p* < 0.001), we averaged the two variables into a single prosocial behavior item (as the two scales for “willingness to help” were different, the two items were standardized prior to being averaged).

##### Manipulation check

The participants were then asked to rate seven items which were sad, sympathy, warm, pity, moving, soft-hearted, and lonely using a 7-point scale from 1 (absolutely not) to 7 (very strong). The rating of the item “lonely” was the index of the manipulation of loneliness, and the ratings of the other six items were indices of whether the manipulations of situation and loneliness also induce other moods.

### Results

The datasets were analyzed using SPSS 18.0; the descriptive analyses are listed in Table 3. To check the experimental manipulations, seven parallel 2 (loneliness: lonely vs. non-lonely) × 2 (situation: public vs. private) analyses of variance were conducted. First, for the six parallel analyses of variance that were conducted to check the current moods of the participants (sad, sympathy, warm, pity, moving, and soft-hearted), the data revealed that the priming of loneliness and situation did not alter the participants' moods (all *F* < 1.0, and all *p* > 0.05). These analyses suggest that the results of Study 2 cannot be explained by a participant's bad mood or feelings of empathy. In addition, for the analysis of variance which made the current feeling of loneliness as the independent variable, only the main effect of the loneliness manipulation was significant [*F*_(1, 164)_ = 5.32; *p* < 0.02; $\eta_{p}^{2}$ = 0.031]. In contrast, we found neither a significant main effect for situation manipulation, nor an interactive effect between situation manipulation and loneliness priming. These results show that the participants felt lonelier in the loneliness priming group than in the non-loneliness priming group.

**Table 3:** **Means (standard deviations) of the willingness to offer financial and time assistance by publicness, loneliness, and gender in Study 2**.

	**In public**		**In private**	
	**Lonely**	**Non-lonely**	**Lonely**	**Non-lonely**
**FEMALES**				
Money	3.16 (1.10)	2.68 (0.87)	3.14 (1.08)	2.72 (0.94)
Time	3.03 (1.11)	2.41 (1.21)	2.50 (1.10)	3.00 (1.61)
Prosocial behavior	0.27 (0.85)	−0.27(0.74)	0.06 (0.82)	0.25 (0.81)
**MALES**				
Money	2.19 (0.40)	2.87 (1.25)	2.50 (1.02)	3.19 (0.91)
Time	2.13 (0.96)	2.73 (1.71)	1.79 (1.25)	2.69 (1.54)
Prosocial behavior	−0.57 (0.41)	0.11 (0.95)	−0.16 (0.89)	0.25 (0.86)

**Figure 1:**
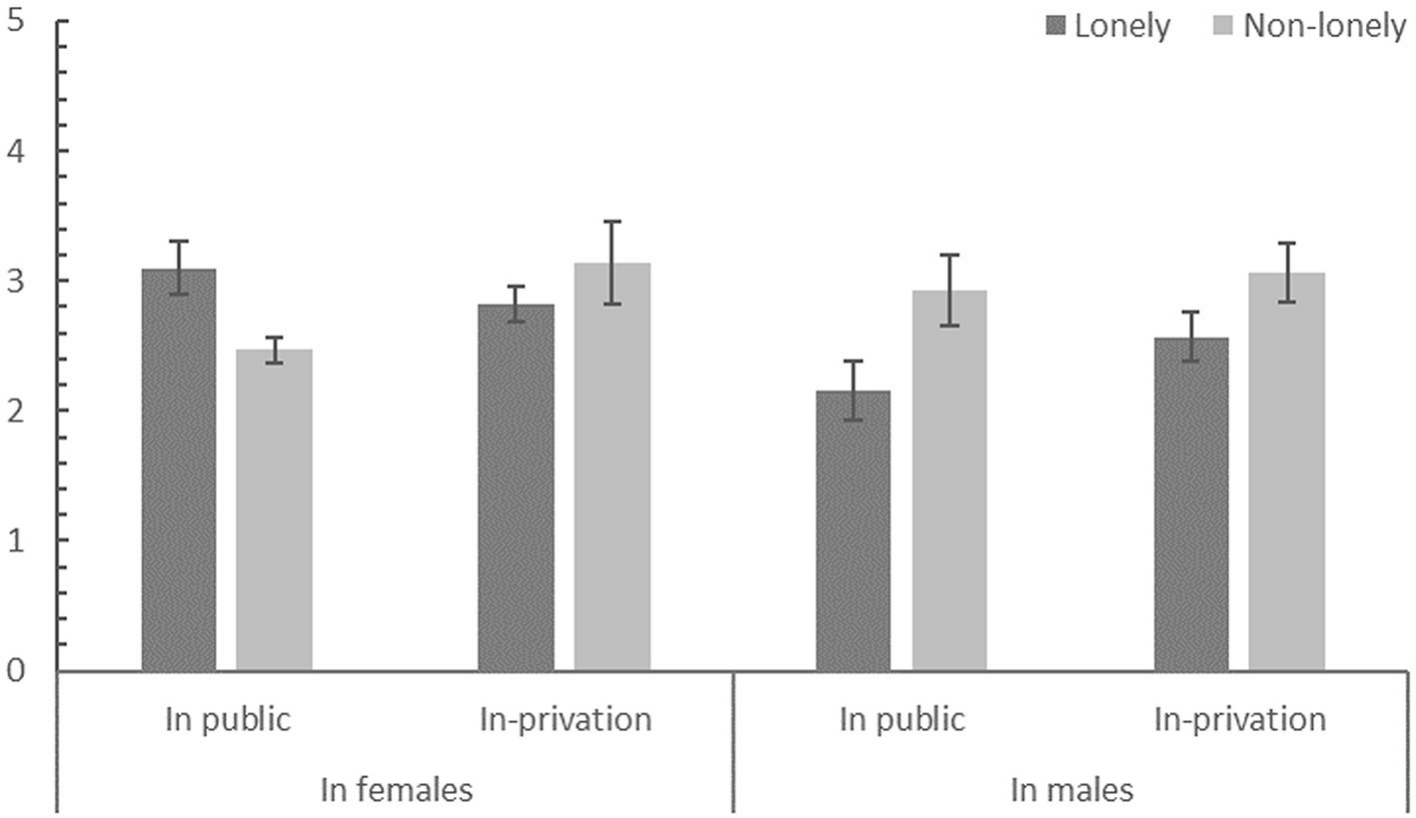
**The three-way interaction effect of situation, loneliness, and gender in prosocial behavior**.

A three-way analysis of variance was conducted with the degree of prosocial willingness as the dependent variable and with situation, loneliness, and gender as independent variables (see Figure 1). The results revealed a significant three-way interaction among gender, situation and loneliness [*F*_(1, 169)_ = 4.45; *p* = 0.036; $\eta_{p}^{2}$ = 0.026]. The following simple-effect analysis revealed that, for females, there was a significant interaction between the public situation and loneliness [*F*_(1, 112)_ = 7.53; *p* = 0.007; $\eta_{p}^{2}$ = 0.063]. When in public, the lonely women were more willing to donate money and time than the non-lonely women were [*t*_(67)_ = 2.88; *p* = 0.005; *d* = 0.543]. However, when in private, the lonely women's willingness to conduct prosocial behavior was equal to the non-lonely women [*t*_(45)_ = 1.15; *p* = 0.24]. For males, only the effect of loneliness was significant [*F*_(1, 57)_ = 7.09; *p* = 0.010; $\eta_{p}^{2}$ = 0.111]. The lonely men were less willing to conduct prosocial behavior than their counterparts [*t*_(59)_ = 2.74; *p* = 0.008; *d* = 0.630].

### Discussion

The findings of Study 2 were similar to the results of Study 1: Study 2 also found a negative role of loneliness in most situations of prosocial behaviors, and a specific positive role of loneliness in prosocial behavior was found only in the public situation.

These results suggest that the effect of loneliness on prosocial behavior is complicated. As previous research has revealed, loneliness destroys prosocial behavior (Salovey et al., 1991; Cassidy and Asher, 1992; Twenge et al., 2007; Woodhouse et al., 2012). However, the results of Study 2 suggest that loneliness may serve the function of motivating individuals to use prosocial behavior to reconnect with other individuals, especially in the public situation, in which the lonely individual's effort to reconnect with others may be best rewarded. The individual difference was not due to a participant's bad mood or the feeling of empathy.

There is a continuing debate about whether the nature of prosocial behavior is selfish or altruistic (Barragan and Dweck, 2014). The results of Study 2 imply that public prosocial behavior may be seen as self-serving and egotistically motivated, since people who only give in public are likely to be seeking acknowledgement or praise. This result is in accordance with the results of Arfer et al.'s research (2015), which found that participants were more prosocial when they were told that their partner would see their choices. Accordingly, they conclude that reputational concerns are a key restraint on selfish exploitation under moral hazard.

Moreover, similarly to the results of Study 1, the findings of Study 2 also suggest a gender difference in the pattern of how loneliness and publicness affect prosocial behavior. The effect of loneliness in promoting prosocial behavior appears to be more robust in women than in men. This finding is similar to some previous studies; for example, while the lonely females appeared more cooperative than the non-lonely females, the lonely males were less cooperative than the non-lonely males (Hanson and Jones, 1981).

## General discussion

The theoretical frameworks concerning loneliness contain two conflicting perspectives on how people respond when fundamental belonging needs are not met: the *loneliness-reduction perspective* and the *loneliness perpetuation perspective* (Vanhalst et al., 2015). Using two studies, the present research indicates that loneliness, both dispositional and experimentally manipulated, plays complicated and conflicting roles in people's prosocial behavior. It seems that whether an individual will respond in a manner consistent with a *loneliness-reduction* dynamic or a *loneliness-perpetuation* dynamic mostly depends on whether the situation is public or private, and this effect is more obvious in women.

First, the results of our research can also be understood from the perspective of the interpersonal nature of prosocial behavior. Although, previous theories suggested two motivations for prosocial behavior (the pure altruism motivation and the egoistic motivation; Andreoni, 1990), the results of the present research give support to the latter theory. Undeniably, there is plenty of evidence showing that the donation behavior which occurs in more private or anonymous situations can still have the power to enhance positive feelings and feelings of social connectedness (e.g., Andreoni, 1990; de Waal and Suchak, 2010). However, this does not mean that this prosocial behavior is necessarily purely altruistic. Recent research reveals that the “pure altruism” model lacks predictive power. In fact, there are ubiquitous trade-offs between the two competing motivations which may be shaped by the evolution of the species. In a certain situation, the trade-offs may be affected by factors such as the openness or the feeling of the individual, with the ultimate goal of improving human survival (Hoffman et al., 2016). From this perspective, prosocial behavior is also a useful interpersonal tool to adapt to the social environment (Leary and Allen, 2011). To adapt to the social environment, the individual must be perceived as the type of person who is a desirable group member, friend, or team member. Because being prosocial may be central to relational value, people are motivated to foster desired images, and thus many of the actions that people take to establish, promote, and maintain belonging involve a strong self-presentational feature (Leary, 1995; Arfer et al., 2015). When people are motivated to be accepted by others, they want to present images of themselves that indicate that they are more rather than less likeable and supportive of the group or relationship. Sometimes, individuals may merely make verbal claims about themselves that convey these desired images, as the results of Study 1 suggested. However, people often must demonstrate certain behaviors to show that they are the type of person with whom others will value having social relationships, as Study 2 suggested. From this perspective, our research also supports the selfish altruism hypothesis (Burnstein, 2005), which claims that prosocial actions may also be driven by egoistic and self-centered concerns (e.g., Cialdini et al., 1997).

Second, the present research demonstrates that loneliness does not necessarily decrease the tendency toward prosocial behavior; instead, loneliness promotes prosocial behavior in the public situation. This result is in accordance with some previous studies. For example, Jiao and Wang (2013) found that lonely people are more likely than non-lonely people to make a moral utilitarian choice, and it may be the desire for connection that leads lonely people to choose more utilitarian choices than non-lonely people do. Moreover, Mead et al. (2011) found that socially excluded people are more likely to sacrifice their personal wellbeing for the sake of social inclusion; for example, socially excluded people reported more willingness to try an illegal drug than did non-excluded people. Recent research in neuroscience found that lonely individuals show increased activity in the ventral striatum, a reward-related region, which reflects the increased desire for social connection (Inagaki et al., 2015). Our results add to the understanding of the interpersonal nature of loneliness. Human beings are social creatures. Similar to pain, hunger, and thirst, loneliness is a signal that alerts individuals to something that is essential for our survival. The threat and pain of loneliness prompts humans to renew connections, and using prosocial behavior is an effective strategy to achieve this goal, as such behavior can establish or enhance one's relational value or attachment with others (Givertz et al., 2013). From this perspective, loneliness is a built-in cue for humans, motivating them to connect with others (Hawkley and Cacioppo, 2010).

Our study also found gender difference in the relationship between loneliness and prosocial behavior. It seems that a seemingly unaccepting social environment could elicit oppositional behavior from females and males. Similar results were also found in Western culture. For example, while lonely females appeared more cooperative and more responsive than the non-lonely females, the lonely males were less cooperative than the non-lonely males (Hanson and Jones, 1981). It may be because males are more affectively independent of their social connections (Al Khatib, 2012; Haferkamp et al., 2012). Moreover, when being excluded, males are more likely to feel their social environment is unworthy and hostile (Gohier et al., 2013). Our results also give support to the wider hypothesis of significant gender-based differences in mental processing (Maccoby and Jacklin, 1974).

The present research provides valuable insights on how loneliness is related to prosocial behavior. Showing the conflicting effects of loneliness on prosocial behavior adds to our knowledge of both the nature of loneliness and the nature of prosocial behavior. The Chinese sample also adds to our understanding of this issue in non-Western culture. The research by Chen et al. (2014) suggests a culture difference, whereby the level, functions and processes of loneliness may be different in a self-oriented society compared with an other-oriented society. In the future, cross-cultural studies are needed to determine which factors contribute to the difference between Chinese and Western samples. Finally, two important limitations to this study must be mentioned. First, although the processes in both Study 1 and Study 2 were carried out semi-anonymously, and the respondents in Study 1 and the groups in Study 2 were believed equal in socially desirability, the self-reporting nature of the current study means its results may be affected by the socially desirable effect. Second, there may be some possible mediators in the relationship between loneliness and prosocial behavior; for example, depression (Vanhalst et al., 2012). In the future, the potential factors in the effects of loneliness on prosocial behavior should be investigated further.

## Author contributions

HH, YL designed experiments; HH, YL carried out study 1; HH, YL, and XL carried out experiment in study 2; HH, YL analyzed results in study 1; HH and XL. analyzed experimental results in study 2; HH, YL, and XL wrote the manuscript.

### Conflict of interest statement

The authors declare that the research was conducted in the absence of any commercial or financial relationships that could be construed as a potential conflict of interest.
